# The Use of the Acute Physiology and Chronic Health Evaluation II (APACHE II) Score and C-reactive Protein/Albumin Ratio to Predict Morbidity and Mortality in Patients Undergoing Emergency Exploratory Laparotomy

**DOI:** 10.7759/cureus.79014

**Published:** 2025-02-14

**Authors:** Rajguru Siwach, Chintamani Chintamani

**Affiliations:** 1 Department of General Surgery, Vardhman Mahavir Medical College and Safdarjung Hospital, New Delhi, IND

**Keywords:** abdominal sepsis, apache-ii score, crp/albumin ratio, emergency exploratory laparotomy, morbidity and mortality, prognostic marker

## Abstract

Background

Exploratory laparotomy in emergency setting is associated with significant post-operative morbidity and mortality. Several prognostic indices have been developed to predict the outcomes. The present study investigated the APACHE II (Acute Physiology and Chronic Health Evaluation II) score and C-reactive protein (CRP)/albumin ratio as predictors of morbidity and mortality in patients who underwent emergency exploratory laparotomy at a tertiary-care hospital.

Methods

One hundred consecutive patients undergoing emergency laparotomy were enrolled. The APACHE II score and CRP/albumin ratio were calculated. Post-operatively, all complications (Clavien-Dindo classification) and deaths within 30 days of surgery were observed. Comparison of the receiver operating characteristic curve was performed to compare the area under the curve (AUC) of APACHE II and CRP/albumin ratio in predicting complications and mortality.

Results

The APACHE II score ≥13 predicted major complications with a sensitivity of 93% and a specificity of 81%. The CRP/albumin ratio ≥5.1 was found to predict major complications with a sensitivity and specificity of 95% and 93%, respectively. The AUC for the APACHE II Score and CRP/albumin ratio in predicting major complications was 0.946 and 0.97, respectively (p <0.001), thus demonstrating excellent diagnostic performance.

Conclusion

We found that the better parameter in terms of AUC is the CRP/albumin ratio, although statistically there was no significant difference in the diagnostic performance of the two parameters.

## Introduction

A prognostic factor is defined as a variable which can be used to anticipate the chance of recovery from a disease or the chance of disease relapse. Several prognostic indices have been developed to predict the outcome of surgical critically ill patients like “Acute Physiology and Chronic Health Evaluation II” (APACHE II) [1] and Sequential Organ Failure Assessment (SOFA) [2].

APACHE II is the sum of three units: an Acute Physiology Score, a Chronic Health Evaluation, and a score based on patient’s age [3]. It includes 12 physiological variables. The APACHE II score is used within the first 24 hours of hospital admission. The APACHE II score ranges from zero to 71 points: 0 to 60 for physiological variables, 0 to 6 points for age, and 0 to 5 points for chronic health conditions [4]. A higher score corresponds to more severe disease and high mortality.

Various biological markers such as C-reactive protein (CRP), albumin, and blood lactate levels seem to correlate with the degree of inflammation during the immediate post-operative phase and are being increasingly used as independent predictors of morbidity and mortality. CRP is an acute-phase protein synthesized by the liver, levels of which rise in response to inflammation. CRP plasma levels increase from around 1mcg/ml to over 500mcg/ml within 24-72 hours of severe tissue damage (trauma, cancer, etc.) [5]. However, when the stimuli end, CRP values decrease exponentially over 18-20 hours, close to the half-life of CRP [6]. Serum albumin is a negative acute phase marker that is downregulated rapidly by inflammatory signals. A post-operative decrease of serum albumin can be used as a marker of the surgical stress response and an early predictor in the clinical outcome after major surgery [7].

CRP is a positive inflammatory marker, whereas albumin is a negative inflammatory marker. Combining the two tests can increase the sensitivity of the test. It has been shown that the ratio between CRP and albumin correlates positively with infection. CRP/Albumin ratio>2 presented the highest sensitivity and specificity in the prediction of 90-day mortality in patients with sepsis/septic shock [8]. None of the indices have 100 percent sensitivity and 100 percent specificity. Furthermore, the CRP/albumin ratio is easy to do and compared to APACHE II does not require the collection of multiple parameters. There are very few studies comparing the prognostic value of the CRP/albumin ratio and APACHE II in patients undergoing emergency exploratory laparotomy. Therefore, this study was conducted to compare APACHE II and CRP/albumin ratio in predicting morbidity and mortality in patients undergoing emergency exploratory laparotomy.

## Materials and methods

Study details

This study was conducted in the Department of General Surgery, Vardhman Mahavir Medical College and Safdarjung Hospital, New Delhi. One hundred consecutive patients admitted to the emergency department during the study period requiring abdominal surgery were evaluated and included in the study. This is an observational cohort study conducted for 18 months (December 1, 2019 to May 31, 2021). The sample size was 100.

Sample size calculation

In the study by Basile-Filho et al. [9], it was observed that the area under the curve (AUC) of APACHE II and CRP/albumin ratio in predicting mortality was 0.85 and 0.731, respectively. Taking this value as reference, δ as .075, and 5% level of significance, the calculated sample size was 92 patients. To reduce the margin of error, the total sample size taken was 100.

The formula used was $n = \frac{(1 - \text{AUC})}{2} &times; \left( \frac{Z_{\alpha/₂}}{&delta;} \right)^{&sup2;}$

Where Zα is the value of Z at a two-sided alpha error of 5% and δ is .075

Calculations

Calculations for the sample size are as per the formula given, using the results of the reference study discussed above.

APACHE II- n ≥ (1- 0.85)/2) × (1.96/.075)2=51.22= 52 (approx.)

CRP/Albumin- n ≥ (1-0.731)/2) × (1.96/.075)2=91.86= 92 (approx.)

As the sample size should be n ≥ 92, the sample size was taken as 100.

Pre-operative assessment

After admission, a detailed clinical history of the patient was recorded which included age, duration of symptoms, and past medical or surgical illness. The patients were then subjected to a thorough clinical examination which included vitals and general, physical, and abdominal examination. All appropriate investigations were done for the patients. Following the initial assessment, simultaneous resuscitation was started. Disease severity was calculated as per the APACHE II score. The APACHE II was calculated on admission. CRP, serum albumin, and their ratio were measured within 24 hours of surgery.

After this evaluation, patients undergoing emergency exploratory laparotomy were enrolled in the study as per the inclusion and exclusion criteria (Table 1) after informed consent. The patients were taken up for surgery after adequate resuscitation.

**Table 1:** Inclusion and Exclusion Criteria

Inclusion criteria	Exclusion criteria
All patients >18 years undergoing emergency exploratory laparotomy	Patients with enterocutaneous or pancreato-biliary fistula
	Patients operated outside
	Pregnant patients

Post-operative outcomes

A detailed assessment of the post-operative outcomes was made. All complications (Clavien-Dindo classification) and deaths within 30 days of surgery were observed. Grade III & IV complications as per Clavien-Dindo classification were considered major complications.

Statistical analysis

Categorical variables were presented in number and percentage (%) and continuous variables were presented as mean ± standard deviation (SD) and median. The receiver operating characteristic (ROC) curve was used to find out the area under the curve of APACHE II and CRP/Albumin ratio for predicting mortality. Diagnostic tests were used to assess sensitivity, specificity, PPV, and NPV. The Mcnamer test was used to compare sensitivity and specificity. Comparison of the ROC was performed to find any significant difference in the AUC of APACHE II and CRP/Albumin ratio. A p-value of <0.05 was considered statistically significant. The data was entered in an MS Excel spreadsheet and analysis was done using IBM SPSS Statistics for Windows, Version 21 (Released 2012; IBM Corp., Armonk, New York, United States).

## Results

**Figure 1:**
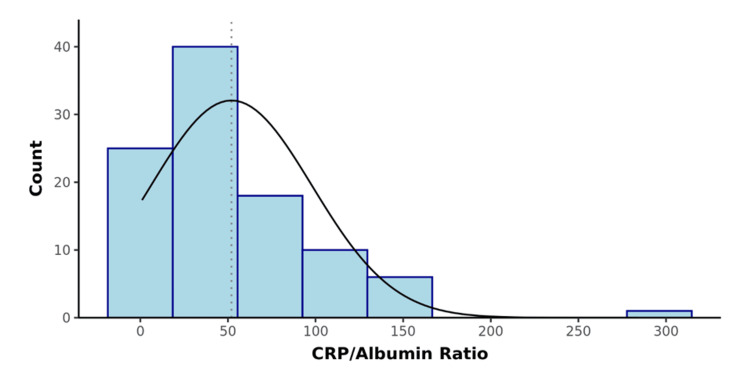
Distribution of the CRP (mg/L)/Albumin (g/dL) Ratio (n=100)

**Figure 2:**
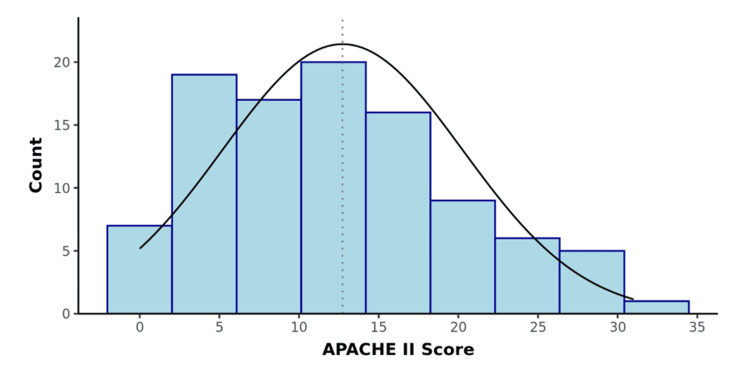
Distribution of the APACHE II Score (n=100) APACHE II: Acute Physiology and Chronic Health Evaluation II

**Figure 3:**
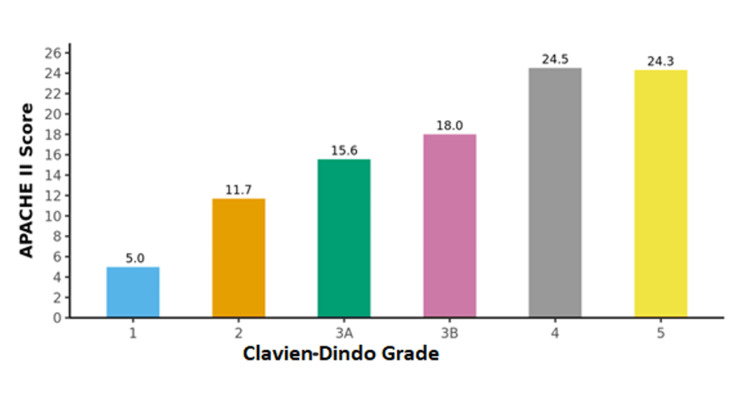
Association between the Clavien-Dindo Classification and APACHE II Score APACHE II: Acute Physiology and Chronic Health Evaluation II

**Figure 4:**
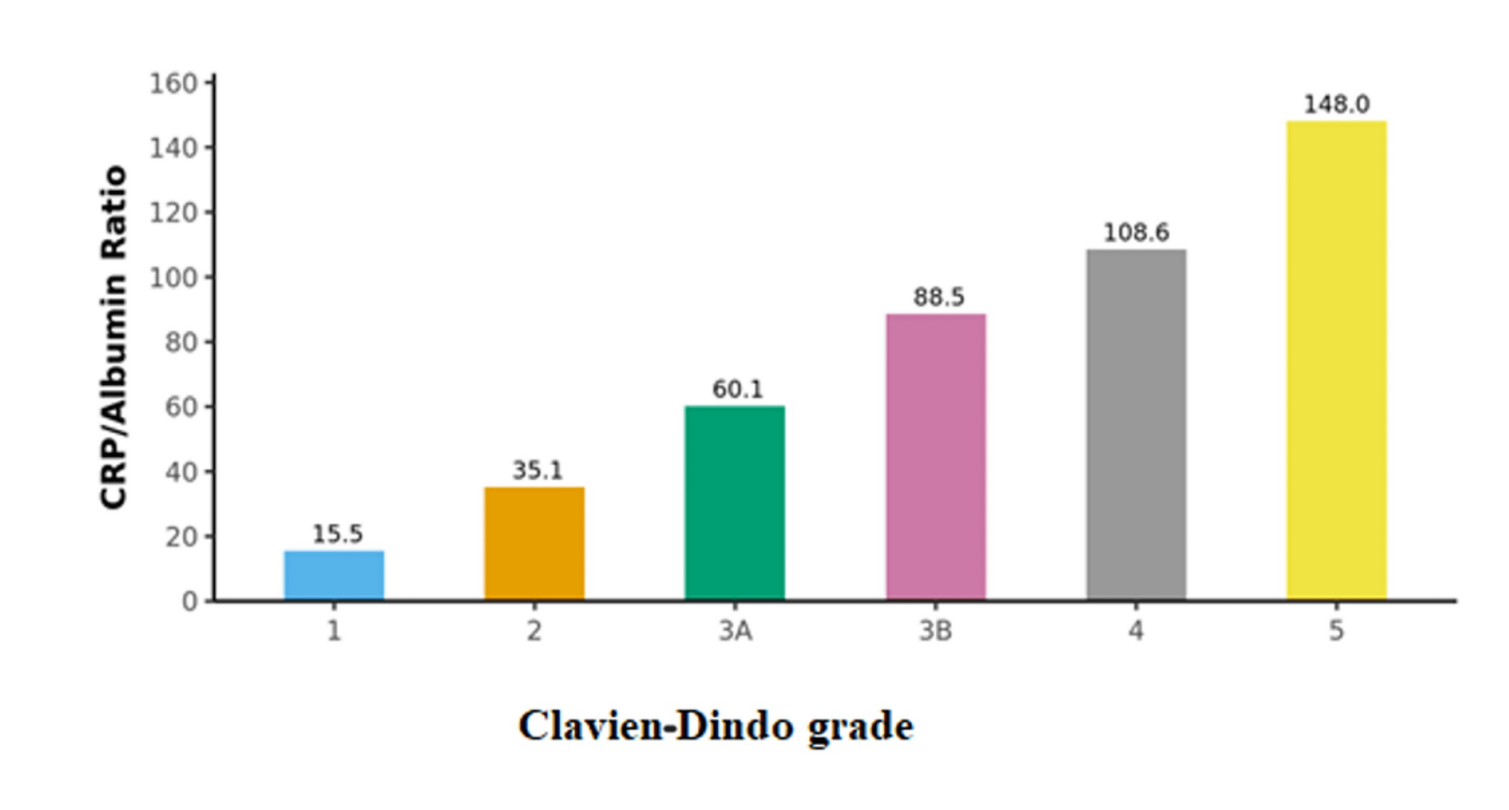
Association between Clavien-Dindo Classification and CRP (mg/L)/Albumin (g/dL) Ratio

A total of 100 participants were recruited for the study as per the inclusion criteria. The CRP/albumin ratio and APACHE II score were calculated for all the patients. The distribution of participants according to the CRP/albumin ratio and APACHE II score was determined (Table 2, Figures 1, 2). The surgical outcomes were graded as per Clavien-Dindo classification and association with the prognostic scores was studied. There was a significant difference between the outcome groups in terms of APACHE II (χ2 = 79.967, p <0.001) and CRP/Albumin ratio (χ2 = 84.248, p = <0.001), with the median values being highest in Grade 5 (Figures 3, 4).

**Table 2:** Distribution of the Participants in Terms of CRP (mg/L), Albumin (g/dL), CRP/Albumin Ratio, and APACHE II (n = 100) IQR: Interquartile range; APACHE II: Acute Physiology and Chronic Health Evaluation II

Parameter	Mean (SD)	Median (IQR)	Range
CRP (mg/L)	107.59	93.00	3.1 – 398
Albumin (g/dL)	2.32	2.30	1.4 - 3.8
CRP/Albumin	52.02 (46.06)	35.5 (18.75-67)	1 – 284
APACHE II	12.73 (7.55)	12 (6-17)	0 – 31

**Figure 5:**
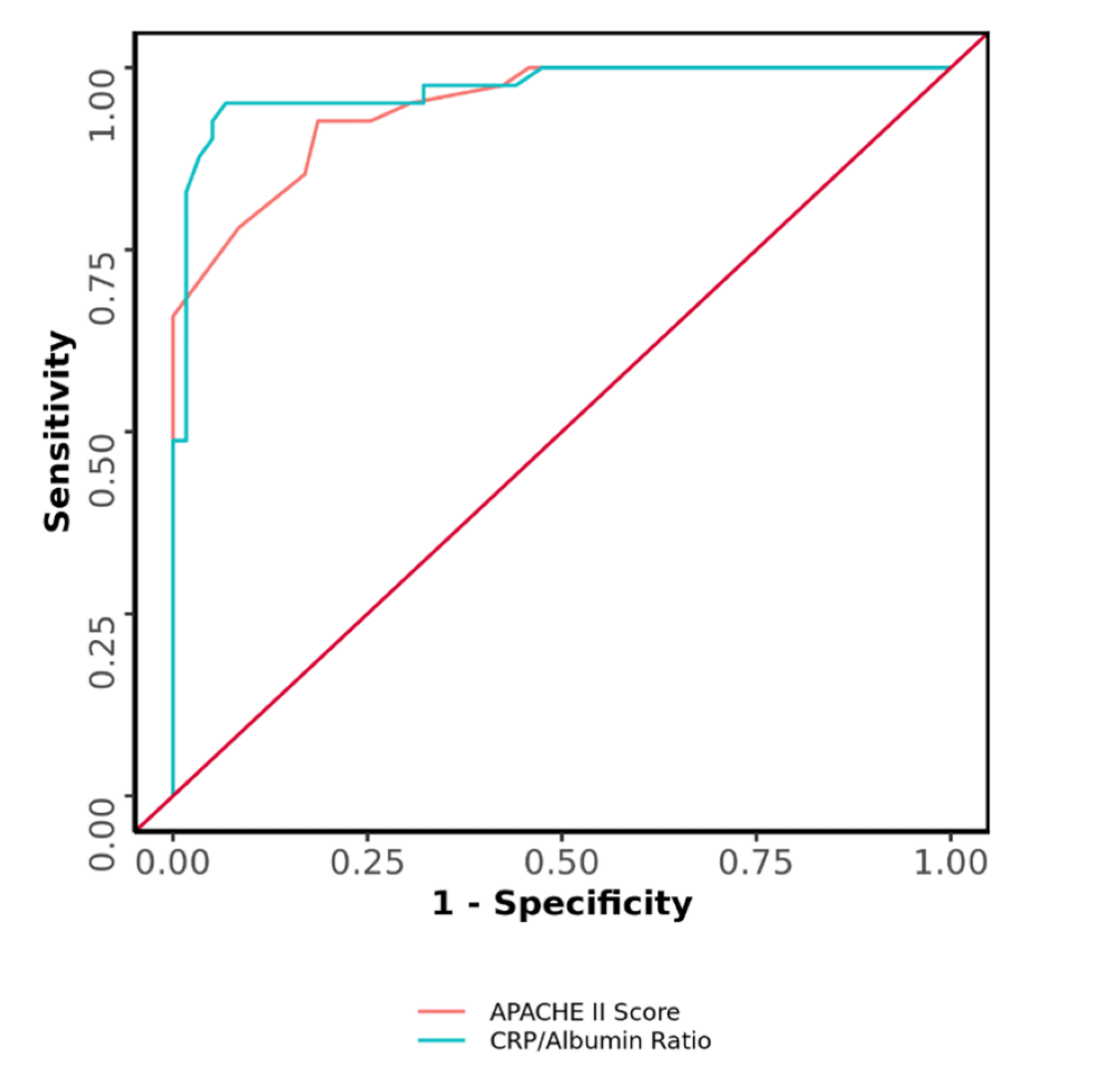
Comparison of the Diagnostic Performance in Predicting Major Complications APACHE II: Acute Physiology and Chronic Health Evaluation II

The AUC for the APACHE II score and CRP/Albumin ratio for predicting major complications was 0.946 (95% CI: 0.907 - 0.985) and 0.97 (95% CI: 0.939 - 1), respectively, thus demonstrating excellent diagnostic performance (p = <0.001). There was no significant difference in the diagnostic performance of the CRP/Albumin ratio and APACHE II score (Table 3, Figure 5).

**Table 3:** Comparison of the Diagnostic Performance of Various Predictors in Predicting Major Complications (n=100) AUC: Area under the curve; CI: Confidence interval; P: P value; Sn: Sensitivity; Sp: Specificity; PPV: Positive predictive value; NPV: Negative predictive value; DA: Diagnostic Accuracy; APACHE II: Acute Physiology and Chronic Health Evaluation II.

Predictor	AUC	95% CI	P	Sn	Sp	PPV	NPV	DA
APACHE II Score	0.946	0.907-0.985	<0.001	93%	81%	78%	94%	86%
CRP/Albumin Ratio	0.97	0.939-1	<0.001	95%	93%	91%	96%	94%

**Figure 6:**
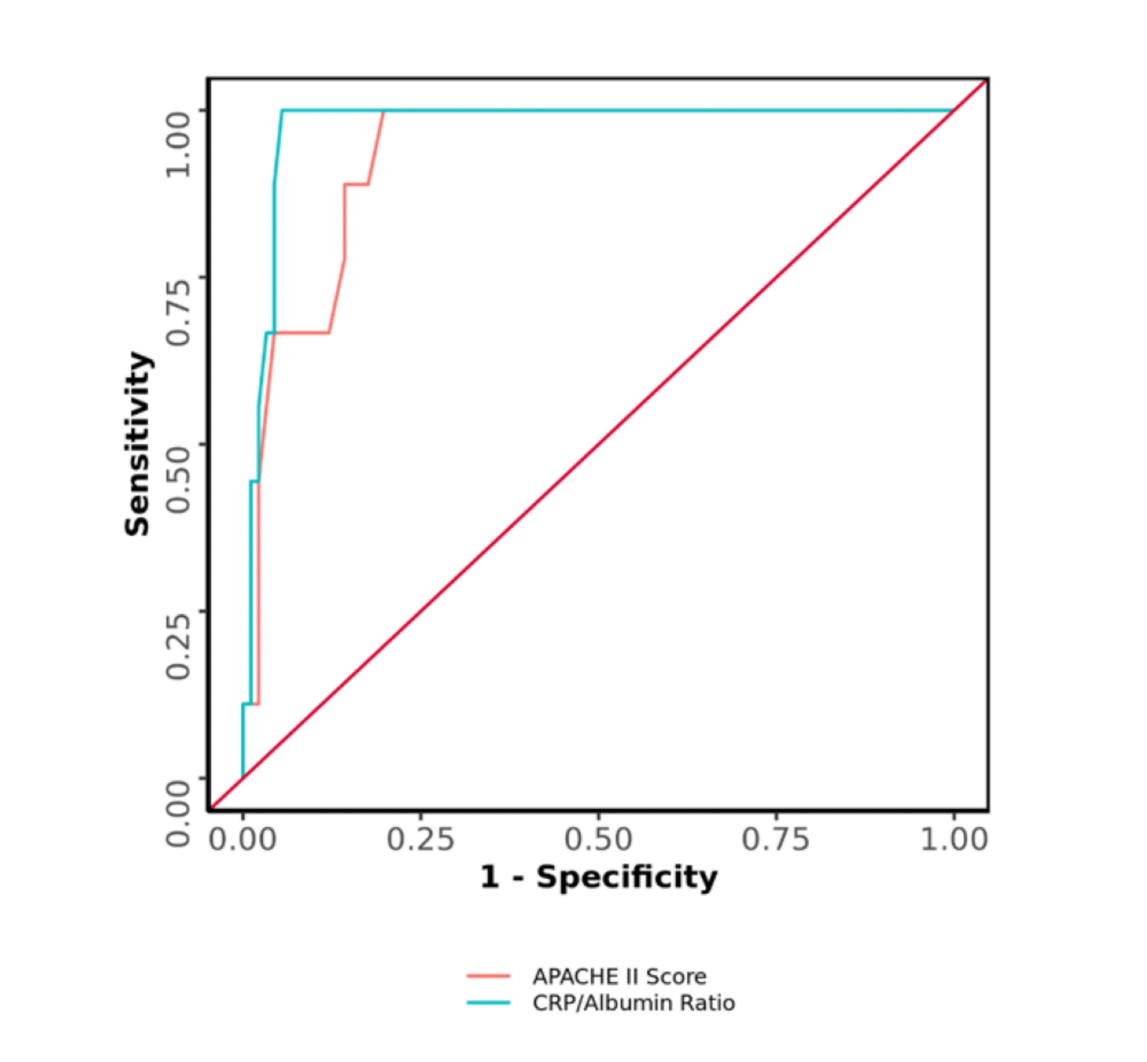
Comparison of the Diagnostic Performance in Predicting Mortality

The AUC for the APACHE II score and CRP/Albumin ratio for predicting mortality was 0.934 (95% CI: 0.878 - 0.991) and 0.976 (95% CI: 0.948 - 1), respectively, thus demonstrating excellent diagnostic performance (p = <0.001). There was no significant difference in the diagnostic performance of the CRP/Albumin ratio and APACHE II score (Table 4, Figure 6).

**Table 4:** Comparison of the Diagnostic Performance of Various Predictors in Predicting Mortality (n=100) AUC: Area under the curve; CI: Confidence interval; P: P value; Sn: Sensitivity; Sp: Specificity; PPV: Positive predictive value; NPV: Negative predictive value; DA: Diagnostic Accuracy; APACHE II: Acute Physiology and Chronic Health Evaluation II.

Predictor	AUC	95% CI	P	Sn	Sp	PPV	NPV	DA
APACHE II Score	0.934	0.878-0.991	<0.001	100%	80%	33%	100%	82%
CRP/Albumin Ratio	0.976	0.948-1	<0.001	100%	94%	64%	100%	95%

A cut-off of APACHE II score ≥13 predicts major complications with a sensitivity of 93%, and a specificity of 81%. A cut-off of APACHE II score ≥17 predicts mortality with a sensitivity of 100%, and a specificity of 80%. The relative risk (95% CI) for mortality when the APACHE II score is ≥17 was 25.33 (4.34-151.36).

A cut-off of CRP/Albumin ratio ≥51 predicts major complications with a sensitivity of 95% and a specificity of 93%. A cut-off of CRP/Albumin ratio ≥110 predicts mortality with a sensitivity of 100% and a specificity of 94%. The relative risk (95% CI) for mortality when the CRP/Albumin ratio is ≥110 was 58.67 (10.29-340.94).

## Discussion

The study was conducted in patients undergoing emergency exploratory laparotomy in a tertiary care hospital. The aim of the study was to use and compare the APACHE II score and CRP/Albumin ratio in predicting morbidity and mortality in such patients. A total of 100 patients following the pre-defined inclusion criteria were recruited. Approximately 50% of the patients were in the age group 21-40 years. There was male preponderance in the study with 84% of the participants being male. Other similar studies conducted also had male preponderance [10,11]. Patients who came to the surgical emergency department of Safdarjung Hospital were diagnosed and prepared for surgery for various reasons including hollow viscus perforation peritonitis, acute small and large intestinal obstruction, gangrenous bowel, ruptured liver abscess and blunt and penetrating trauma to the abdomen with hemoperitoneum, etc. underwent emergency exploratory laparotomy with needful procedure under general anesthesia based on underlying pathology.

On admission, the APACHE II prognostic score and CRP/Albumin ratio were calculated. In our study, the mean APACHE II score was 12.73 and it ranged from 0 to 31. The CRP/Albumin Ratio had a mean value of 5.20 (4.60) and ranged from 0.1 to 28.4.

On classifying the patients based on post-operative outcomes (Clavien-Dindo classification), 59.0% of the participants had minor complications (grade 1 and grade 2), 32.0% of the participants had major complications (grade 3A, 3B and grade 4) and 9.0% of the participants succumbed to death (grade 5). Hansted et al. [12] in their study on 885 patients undergoing emergency abdominal surgeries recorded all-cause 90-day mortality to be 8.9% which was similar to that noticed in our study.

It was found that APACHE II Score ≥13 predicts major complications with a sensitivity of 93% and specificity of 81%. A cut-off of APACHE II Score ≥17 predicts mortality with a sensitivity of 100% and specificity of 80%. In a study by Capoor et al. on patients with enteric perforation, an APACHE II score >13 was associated with 46.2% mortality and 100% morbidity [13]. In a study by Kulkarni et al. [14] on 50 patients with peritonitis due to hollow viscus perforation, the mean APACHE II score in survivors was 9.88, whereas in non-survivors it was 19.25. We also found a significantly higher mean APACHE II score in non-survivors (24.3) as compared to survivors (11.58).

On analysis of the CRP/albumin ratio in different outcomes based on Clavien-Dindo classification, The mean of CRP (mg/L)/Albumin(g/L) in the non-survivor group and survivor group was 14.8 and 4.25 respectively. A cut-off of CRP/Albumin Ratio ≥5.1 was found to predict major complications with a sensitivity and specificity of 95% and 93% respectively. A cut-off of CRP/Albumin Ratio ≥11 predicts Mortality with a sensitivity and specificity of 100% and 94% respectively.

Ranzani et al. conducted a study on 334 patients to predict the 90-day survival post ICU discharge and found lower survival rates in patients with CRP/albumin ratios >2 at the time of discharge from ICU [15]. Swarnkar et al. found that patients undergoing major abdominal surgeries with a CRP/Albumin ratio value of more than 2.16 on post op day 3 had higher risk of post op complications [16].

The AUC for the APACHE II score and CRP/Albumin ratio predicting major complications was 0.946 (95% CI: 0.907 - 0.985; p <0.001) and 0.97 (95% CI: 0.939 - 1; p <0.001), respectively, thus demonstrating excellent diagnostic performance. The AUC for the APACHE II score and CRP/Albumin ratio predicting mortality was 0.934 (95% CI: 0.878 - 0.991; p<0.001) and 0.976 (95% CI: 0.948 - 1; p<0.001), respectively. In a similar study conducted by Basile-Filho et al., the AUC for APACHE II and CRP/Albumin ratio was 0.85 and 0.73, respectively. They concluded that APACHE II is better than the CRP/Albumin ratio in predicting post-operative complications and mortality in sepsis patients [9].

We found that both APACHE II and the CRP/Albumin ratio significantly predict morbidity as well as mortality. It can also be said that a better parameter in terms of AUC is the CRP/Albumin ratio although statistically there was no significant difference in the diagnostic performance of the two parameters in terms of predicting morbidity and mortality.

A small sample size and time constraints are the possible limitations of our study. Further multi-center studies with a larger sample size are required to compare the two parameters.

## Conclusions

We conclude that both the APACHE II score and CRP/Albumin ratio can be used as prognostic markers for the prediction of morbidity and mortality in patients undergoing emergency exploratory laparotomy with significant sensitivity and specificity.

In our study, the CRP/Albumin ratio came out to be the better parameter for the assessment of prognosis; however, the difference was not statistically significant. Further studies are required for comparison of the two parameters for prognostication.
